# Expression of Two RpoH Sigma Factors in *Sinorhizobium meliloti* upon Heat Shock

**DOI:** 10.1264/jsme2.ME17087

**Published:** 2017-12-02

**Authors:** Hisayuki Mitsui, Kiwamu Minamisawa

**Affiliations:** 1 Graduate School of Life Sciences, Tohoku University Katahira, Aoba-ku, Sendai 980-8577 Japan

**Keywords:** α-proteobacteria, heat shock protein, root-nodule symbiont, sigma factor, *Sinorhizobium meliloti*

## Abstract

The plant symbiotic α-proteobacterium *Sinorhizobium meliloti* has two RpoH-type sigma factors, RpoH1 and RpoH2. The former induces the synthesis of heat shock proteins and optimizes interactions with the host. Using a Western blot analysis, we examined time course changes in the intracellular contents of these factors upon a temperature upshift. The RpoH1 level was relatively high and constant, suggesting that its regulatory role in the heat shock response is attained through the activation of the pre-existing RpoH1 protein. In contrast, the RpoH2 level was initially undetectable, and gradually increased. These differential patterns reflect the functional diversification of these factors.

Upon exposure to stresses including heat, cells of virtually all living organisms transiently increase the synthesis of a set of heat shock proteins (hsps), which are typically molecular chaperones and proteases. This response, known as the heat shock response, minimizes damage from thermal denaturation and the aggregation of proteins. In the γ-proteobacterium *Escherichia coli*, an RNA polymerase holoenzyme containing the sigma factor σ^32^ (the *rpoH* gene product) transcribes many of the hsp genes (7). Following a sudden shift from 30°C to 42°C, the σ^32^ level increases approximately 17-fold by 6 min (the initial phase) and then drops to 5 times that at 30°C by 15 min (the adaptation phase); the transcription rate of the σ^32^ regulon member *dnaKJ* changes in parallel with the change in the σ^32^ level (29). The synthesis of σ^32^ is mainly modulated at the translational level (14). In addition, the σ^32^ protein is unstable during steady-state bacterial growth (half-life: approx. 1 min at 30°C) due to proteolytic degradation mediated by the chaperone systems DnaK/DnaJ/GrpE and GroEL/GroES and the ATP-dependent protease FtsH, all of which belong to the σ^32^ regulon. Upon heat shock, misfolded or aggregated proteins titrate these chaperones away, which results in transient σ^32^ stabilization (6, 28, 29, 32). The same chaperones also inactivate the σ^32^ protein in the adaptation phase of the heat shock response (6, 33).

Homologs of σ^32^, referred to here as RpoHs, are present in diverse proteobacteria (16). Similar to *E. coli* σ ^32^, α-proteobacterial RpoHs control the expression of hsps (9, 17, 19, 24, 34). Notably, some α-proteobacteria have more than one RpoH species, which are sometimes not functionally equivalent (4, 11, 20, 22, 23). Two RpoHs, RpoH1 and RpoH2, are encoded in the genome of the α-proteobacterium *Sinorhizobium meliloti*, which lives either as a saprophyte in soil or as a root-nodule nitrogen-fixing symbiont of alfalfa (22, 23). In wild-type *S. meliloti*, the synthesis of several hsps peaks 5–10 min after a temperature upshift, whereas this induction is abolished in a mutant lacking *rpoH1* (23). More than 300 genes have been identified as RpoH1 regulon members by transcription profiling during heat shock (1, 26). In contrast, the *rpoH2* null mutation has no appreciable effects on the heat shock response in either the wild-type or *rpoH1* mutant background (1, 13). Instead, the expression of at least 44 genes depends on RpoH2 in the late stationary phase (1). Notably, the *rpoH1* mutant elicits the formation of nodules with no nitrogen-fixing activity (13, 22, 23). Although the *rpoH2* single mutant has no symbiotic defects, the *rpoH1 rpoH2* double mutant shows more severe defects, including the lack of nodule formation, than the *rpoH1* single mutant (22, 23).

In several α-proteobacteria, RpoHs are transcriptionally up-regulated upon heat shock (19, 20, 24, 34, 35). In some bacteria with multiple RpoH species, the level of each *rpoH* mRNA responds differently to stress (19, 20). In the plant-pathogenic α-proteobacterium *Agrobacterium tumefaciens*, the level of its single RpoH factor increases approximately 5-fold by 15 min of heat shock (17). Notably, activation of the pre-existing RpoH protein is sufficient for the normal induction of hsp synthesis, and the contribution of this increase in the RpoH level is negligible (18).

In the present study, we compared the responses of RpoH1 and RpoH2 levels to heat shock in *S. meliloti* in order to gain insights into the functional diversification of these factors. The bacterial strains and plasmids used are listed in Table 1. The *rpoH1* and *rpoH2* coding regions were amplified by PCR. The primers 5′-TGCCATATGGCCCGCAATACCTTG-3′ and 5′-GGTAAGCTTAGCGCCTTCAACCACGCG-3′ were used for *rpoH1*; the primers 5′-CAACATATGAAGACCCT CACAGCA-3′ and 5′-TACAAGCTTATGCATCGACGCCG TCAG-3′ were used for *rpoH2*. PCR products were cloned into the expression plasmid pET-20b(+); restriction sites used for cloning are underlined. The hexahistidine-tagged recombinant proteins RpoH1-His (calculated molecular mass, 36.1 kDa) and RpoH2-His (33.6 kDa) were produced in *E. coli* BL21(DE3). We prepared antisera against the recombinant proteins. In the Western blot analysis, these antisera recognized authentic RpoH1 (34.6 kDa) and RpoH2 proteins (32.1 kDa) in *S. meliloti* cell extracts; however, the anti-RpoH2 antiserum also reacted with other proteins including RpoH1 (Fig. S1). RpoH2 was only detectable in heat-shocked cells. RpoH1 and RpoH2 were also detected in *S. meliloti* cells isolated from alfalfa nodules (Fig. S1).

Using a Western blot analysis, we examined time course changes in RpoH1 and RpoH2 levels in growing *S. meliloti* cells upon a temperature upshift (from 25°C to 37°C). Protein bands were immunodetected with an ECL Detection System (GE Healthcare, Little Chalfont, England), and images of blots were captured with a LAS-1000plus image analyzer (Fuji Film, Tokyo, Japan). Protein amounts were estimated using calibration curves generated using purified RpoH1-His (1.0–2.5 ng) and RpoH2-His proteins (0.1–2.5 ng). A set of calibration samples was always included along with cell lysate samples in the Western blot analysis. The RpoH1 level was relatively constant before and after the temperature upshift (Fig. 1A). This is consistent with the absence of a significant increase in *rpoH1* transcription upon heat shock (1, 22). The RpoH1 level ranged between 40 and 50 fmol microgram^−1^ of total protein (Fig. 1B), which is markedly higher than that of σ^32^ under non-stress conditions (0.44 fmol microgram^−1^ of total protein at 30°C [5]) and similar to the content of the primary sigma factor σ^70^ (50–80 fmol microgram^−1^ of total protein in cells grown at 37°C [8]) in *E. coli*. In contrast, the RpoH2 level was below the detection limit (<1 fmol microgram^−1^ of total protein) at 25°C, increased gradually after the temperature upshift (up to approx. 5 fmol microgram^−1^ of total protein by 40 min), and remained at this level until 60 min (Fig. 1). The RpoH2 level was significantly higher at 60 min in the *rpoH1* mutant than in the wild type, whereas the RpoH1 level was not significantly affected by the *rpoH2* mutation (Fig. 1B).

The constantly high level of RpoH1 indicates that this protein level is not a limiting factor in hsp synthesis under non-stress conditions, similar to *A. tumefaciens* RpoH (18). The most likely explanation is that RpoH1 is stable but inactive in non-stressed cells, and is activated by heat shock. In *A. tumefaciens*, the DnaK/DnaJ chaperone appears to inactivate RpoH (18). In *S. meliloti*, five genomic loci encode GroEL homologs, of which GroEL5 is induced upon stress in an RpoH1-dependent manner, whereas GroEL1 is present at the highest level and plays a housekeeping role (2, 3, 13). The mutational loss of GroEL1 increases the GroEL5 level in non-stressed cells (3), suggesting that GroEL1 inactivates RpoH1. Studies that assess the half-life of the cellular RpoH1 protein before and after heat shock and examine the effects of engineered reductions in the levels of the major chaperones on the expression of RpoH1-dependent genes will be useful for confirming the above explanation. On the other hand, the increase in the RpoH2 level following heat shock is attributable, at least in part, to an increase in its mRNA level, which is regulated by RpoE2, an extracytoplasmic function sigma factor involved in the general stress response in *S. meliloti* (27). It currently remains unclear whether the higher level of RpoH2 in the *rpoH1* mutant than in the wild type at the late stage of heat shock was caused by further enhanced transcription or a post-transcriptional mechanism such as stabilization of the RpoH2 protein. The sequence similarity between RpoH1 and RpoH2 is lower (41% identical at the amino acid level) than that between RpoH1 and *A. tumefaciens* RpoH (87% identical), and RpoH1 and RpoH2 are similarly distant from *E. coli* σ ^32^ (36–37% identical) (23) (Fig. 2). Thus, analyses of their variants with amino acid substitutions, particularly at the corresponding positions that are critical for chaperone- and FtsH-dependent proteolysis in *E. coli* σ ^32^ (Fig. 2), will provide an insight into the different levels of these RpoHs.

RpoH2 has been reported to not significantly affect the global transcription profile at the early stages of heat shock (1, 13). However, it remains unknown whether accumulated RpoH2 contributes to the induction of a particular set of genes. It is of interest to examine the effects of extended heat shock on the expression of known RpoH2 regulon members, which were identified based on their expression in the stationary phase (1).

## Supplementary Material



## Figures and Tables

**Fig. 1:**
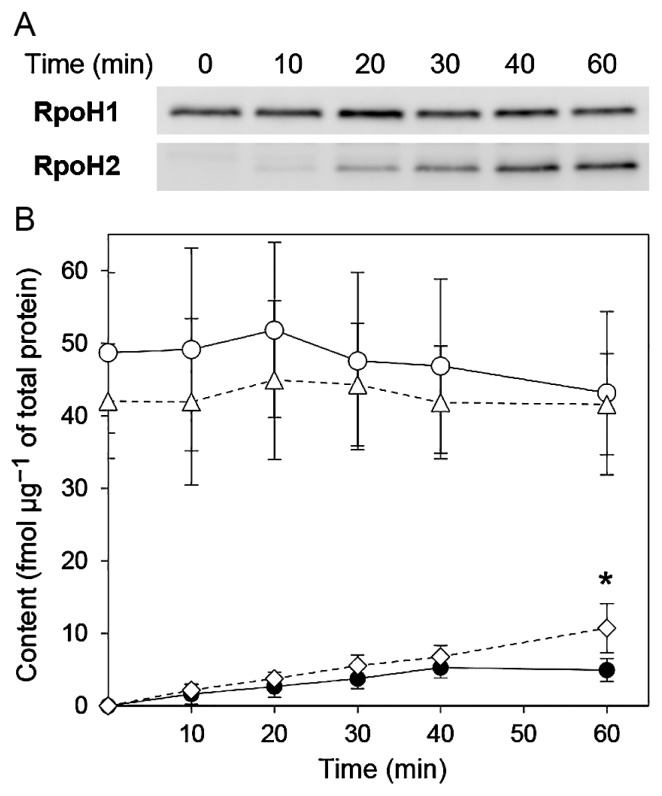
Time course changes in RpoH1 and RpoH2 levels in *Sinorhizobium meliloti* exposed to heat shock. (A) Representative Western blots. Each lane contains lysates of wild-type cells (1 μg total protein for the RpoH1 analysis and 5 μg for the RpoH2 analysis). Cells were grown in LB medium supplemented with MgCl_2_ (2.5 mM) and CaCl_2_ (2.5 mM) at 25°C to an optical density at 660 nm of 0.5 (time 0) and then exposed to 37°C for the indicated time. (B) Quantified data from the Western blot analysis. Contents of RpoH1 in wild-type cells (open circles, solid line) and *rpoH2* mutant cells (triangles, broken line), and those of RpoH2 in wild-type cells (closed circles, solid line) and *rpoH1* mutant cells (diamonds, broken line) were estimated from band intensities. Values are means±SD from at least six measurements. RpoH2 concentrations were significantly different (*t*-test, * *P*<0.001) at 60 min between the wild type and *rpoH1* mutant.

**Fig. 2:**
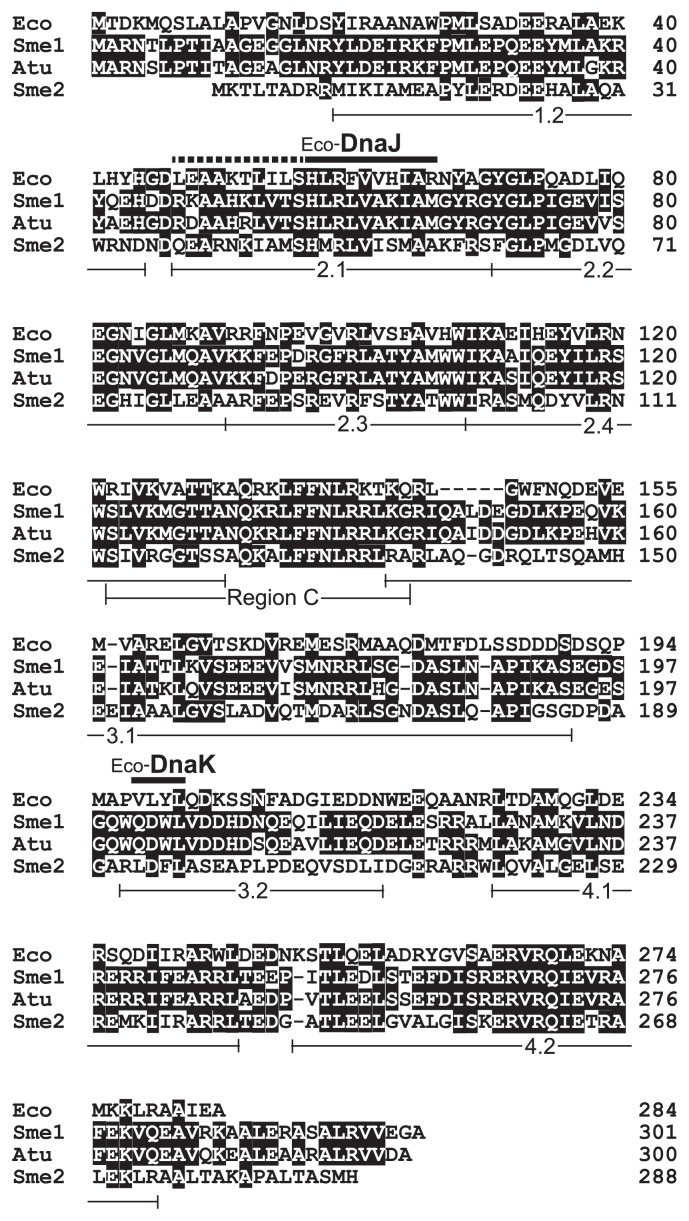
Comparison of amino acid sequences of RpoH-type sigma factors. Eco, *Escherichia coli* σ^32^ (RefSeq accession number NP_417918); Sme1, *Sinorhizobium meliloti* RpoH1 (NP_386832); Atu, *Agrobacterium tumefaciens* RpoH (WP_035228050); and Sme2, *S. meliloti* RpoH2 (NP_387362). Identical residues at each position are shown in white letters on a black background. The DnaJ- and DnaK-binding sites reported for *E. coli* σ^32^ (25) are marked above the alignment; a broken bar (DnaJ) indicates an extension of the binding site proposed later (31). Leu-47, Ala-50, and Ile-54 of σ^32^ may contact the degradation machinery (21). Regions that are highly conserved among all σ^70^-family proteins (regions 1.2 to 4.2) (10) and region C, which is unique to RpoH sigma factors (15), are indicated below the alignment.

**Table 1:** Strains and plasmids used in this study

Strain or plasmid	Characteristics*	Reference or source
*S. meliloti* strains		
Rm1021	Wild type; Sm^r^	12
HY658N	Rm1021 *rpoH1*::*aphII*; Sm^r^ Nm^r^	23
BY294	Rm1021 *rpoH2*::*aacC1*; Sm^r^ Gm^r^	23
*E. coli* strain		
BL21(DE3)	F^−^ *ompT hsdS*_B_(r_B_^−^ m_B_^−^) *gal dcm* λ(*lac*UV5-T7 *gene 1*)	30
Plasmid		
pET-20b(+)	Ap^r^; vector for expression by T7 RNA polymerase	Novagen

* Sm, streptomycin; Nm, neomycin; Gm, gentamicin; and Ap, ampicillin.
