# Supplementary material for: Expression of Two RpoH Sigma Factors in Sinorhizobium meliloti upon Heat Shock
Source: Microbes Environ. 2017 Dec 2;32(4):394–7. doi: 10.1264/jsme2.ME17087 (PMC5745026; doi:10.1264/jsme2.ME17087)
Supplement: Supplementary file 1 [file 32_394_s1.pdf]

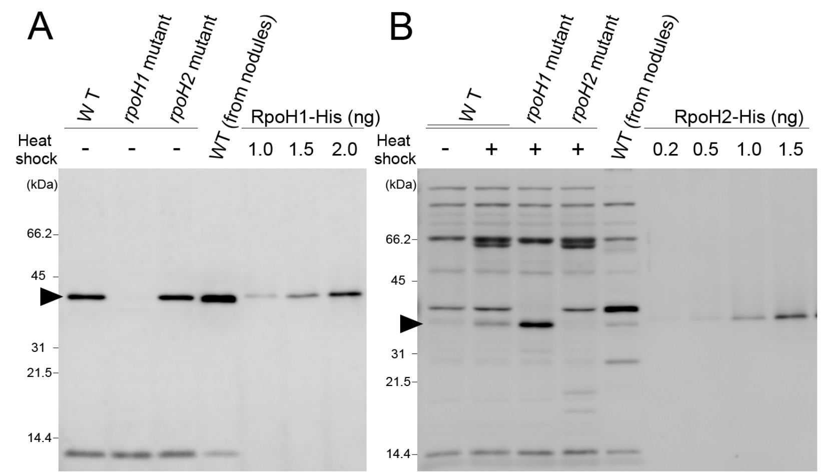

Fig. S1. Western blot detection of RpoH1 and RpoH2 in *Sinorhizobium meliloti* cell lysates. The wild type (WT), *rpoH1* mutant, and *rpoH2* mutant were grown to an OD_{660} of 0.5 at 25°C or were then exposed to 37°C for 60 min in LB medium (10 g tryptone, 5 g yeast extract, and 5 g NaCl per liter; pH 7.0) supplemented with $MgCl_2$ (2.5 mM) and $CaCl_2$ (2.5 mM). Symbiotic nodule bacteria were isolated using Percoll gradient centrifugation (1) from a homogenate of alfalfa nodules formed after inoculation with the wild-type *S. meliloti* strain. Cell lysates (5 µg total protein per lane) and varying amounts of purified RpoH1-His or RpoH2-His protein were subjected to SDS–12.5% polyacrylamide gel electrophoresis. Separated proteins were electroblotted onto a polyvinylidene difluoride membrane (Hybond-P, GE Healthcare), probed with anti-RpoH1 (A) or anti-RpoH2 (B) antiserum, and developed using an ECL Detection System (GE Healthcare). The abundance of RpoH1 in nodule bacteria is attributable to a substantial expression of *rpoH1* throughout the interior nodule tissue (2). Arrowheads indicate the positions of RpoH1 (A) and RpoH2 (B). In both panels, positions of molecular weight markers (unstained SDS-PAGE standards, Bio-Rad) are indicated on the left.

1. Kouchi, H., and K. Fukai. 1989. Rapid isolation of bacteroids from soybean root nodules by Percoll discontinuous gradient centrifugation. Soil Sci. Plant Nutr. 35:301–305.
2. Oke, V., B.G. Rushing, E.J. Fisher, M. Moghadam-Tabrizi, and S.R. Long. 2001. Identification of the heat-shock sigma factor RpoH and a second RpoH-like protein in *Sinorhizobium meliloti*. Microbiology 147:2399–2408.
